# Postpartum glucose follow-up and lifestyle management after gestational diabetes mellitus: general practitioner and patient perspectives

**DOI:** 10.1186/s40200-016-0282-2

**Published:** 2016-12-07

**Authors:** Sarah H. Koning, Helen L. Lutgers, Klaas Hoogenberg, Chris A. Trompert, Paul P. van den Berg, Bruce H. R. Wolffenbuttel

**Affiliations:** 1 Department of Endocrinology, University of Groningen, University Medical Center Groningen, PO Box 30.001, 9700 RB Groningen, The Netherlands; 2 Department of Endocrinology, Medical Center Leeuwarden, Leeuwarden, The Netherlands; 3 Department of Internal Medicine, Martini Hospital, Groningen, The Netherlands; 4 General Practitioners Practice Trompert, Zuidhorn, The Netherlands; 5 Department of Gynaecology and Obstetrics, University of Groningen, University Medical Center, Groningen, The Netherlands

**Keywords:** Gestational diabetes mellitus, Follow-up, Postpartum, Lifestyle, Glucose testing, General practitioner

## Abstract

**Background:**

Incidence of type 2 diabetes is high after gestational diabetes mellitus (GDM). We aimed to evaluate the adherence to follow-up six-weeks postpartum visits in secondary care after GDM and glucose monitoring in primary care longer than 12–14 months after delivery and the years thereafter. In addition, we examined the women’s lifestyle after delivery.

**Methods:**

A cross-sectional follow-up survey among women with a history of GDM and their general practitioners (GP). Rates of attendance at the six-weeks postpartum visit and glucose testing were obtained from hospital records, over the period 2011–2012. Rates of annual follow-up postpartum glucose testing were assessed by a survey among their GP’s. Lifestyle of the women on diet and exercise was assessed by questionnaire in 2015.

**Results:**

In total 197 women were eligible for the study. Of these, 156 (79%) attended the six-weeks postpartum visit at the diabetes outpatient clinic and in 145 (93%) of these women glucose testing was performed. In total 77 (39%) women responded to the invitation to participate in this study and filled in the lifestyle questionnaire. About one third of the women met the recommendations for sufficient physical activity. A majority of them did not fulfil the Dutch guidelines on healthy diet – fruit intake 35.1%, vegetables intake 7.8%. Of the 74 invited GP’s, 61 responded (82%), only 12 (20%) reported that they had performed a follow-up glucose testing within >12–14 months postpartum. Of these women, five were tested only in the first year of follow-up, five also in the second year, and two were tested for three consecutive years.

**Conclusions:**

Despite the high attendance rate of six-weeks postpartum visit and glucose testing, we observed low rates of longer-term follow-up regarding postpartum glucose testing. Moreover, we found a suboptimal adherence to healthy lifestyle for women with a history of GDM.

## Background

Gestational diabetes mellitus (GDM) is historically defined as any degree of glucose intolerance with onset or first recognition during pregnancy [1]. Although in most women with GDM glucose intolerance resolves after delivery, women with a history of GDM are at increased risk of developing impaired glucose tolerance and type 2 diabetes mellitus (DM). It has been estimated that the risk of type 2 DM may be as high as 50–70% in 5–10 years after delivery [2, 3].

Adequate lifestyle interventions may prevent or postpone the development of type 2 DM. In addition, early diagnosis and treatment of type 2 DM may contribute to the prevention of long-term DM complications, including cardiovascular- and renal diseases [4]. Therefore, national and international guidelines recommend follow-up glucose testing in women with a history of GDM [5, 6]. In the Netherlands, the 2010 Dutch Society of Obstetrics and Gynaecology guideline “Diabetes and Pregnancy” recommends glucose testing six weeks after delivery and subsequently once a year for the next 5 years [5]. Our department routinely invited all patients to the Diabetes Centre to attend a six-weeks postpartum visit. Patients then are referred back to their general practitioner (GP), who has a central role in our health-care system and is therefore the most obvious caregiver to perform annual follow-up glucose testing and simultaneously to motivate women to adopt and maintain a healthy lifestyle to prevent type 2 DM.

It is however unclear how well the advices of this guideline are implemented. To improve early diagnosis of type 2 DM after GDM, we should first verify how many GP’s are aware that women with a history of GDM need annual follow-up glucose testing and take direct responsibility for follow-up glucose testing. For instance, do they have a system to track former GDM patients? In addition, it is important to know whether women with a history of GDM are aware of the recommended annual follow-up glucose testing, and take responsibility for visiting their GP once a year out of their own initiative and change their lifestyle. Successful monitoring depends on clear guidelines, good implementation in primary and secondary care, and education of self-management and adherence of the patient.

Hence, we evaluated the adherence-rate of the follow-up postpartum visit in secondary care after GDM and glucose testing longer than > 12–14 months after delivery and the years thereafter in primary care. We also examined by questionnaire the lifestyle of the women with a history of GDM including physical activity and diet.

## Methods

### Study participants

This study is a cross-sectional follow-up survey of former women with GDM and their GP’s. All 215 women who were treated for GDM at the University Medical Center Groningen, with a first visit between January 2011 and December 2012, were eligible for participation.

All pregnant women had GDM screening at week 24–28 of gestation if they had one or more GDM risk factors [5]. The World Health Organization 1999 criteria were used to diagnose GDM (fasting plasma glucose value ≥ 7.0 mmol/l and/or a two-hour value ≥ 7.8 mmol/l after a 75-g oral glucose tolerance test (OGTT)) [7]. In accordance with the standard of care, all women diagnosed with GDM were referred to the dietician for dietary counselling. If after 1–2 weeks the fasting plasma glucose was > 5.3 mmol/l and/or postprandial plasma glucose level > 7.8 mmol/l, insulin therapy was started. Routinely this is stopped after delivery.

Women were invited to visit the outpatient clinic six weeks after delivery. During this visit, laboratory testing for blood glucose values, HbA1c, lipid profile, and microalbumuria was performed and all women received information about the future risk of developing type 2 DM. Glucose values were evaluated with results of a 75-g OGTT, HbA1c and/or self-monitoring of the blood glucose (SMBG) values. Women who were breast-feeding were recommended to perform additional glucose testing after cessation of breastfeeding. Targets for treatment were discussed, including weight reduction and information about healthy lifestyle, and they were verbally instructed to visit their GP at least annually for follow-up glucose testing. The GP received a discharge letter mentioning the increased risk for development of type 2 DM and a formal advise to invite their patient for –at least- annual follow-up glucose testing.

For the present study, all the women treated for GDM between January 2011 and December 2012 were invited, including women who did not visit the six-weeks postpartum glucose visit and/or women who did not test their glucose values at the six-weeks postpartum visit. Women were not invited to participate if they had a still birth (*n* = 2). The GP was invited to fill in a questionnaire when their patient gave informed consent. The study was conducted in accordance with the guidelines of the Declaration of Helsinki and Good Clinical Practice, and approved by the Medical Ethical Review Committee of the University Medical Center Groningen.

### Procedure and data collection

Clinical and demographic data of all eligible women were obtained from the electronic medical records, including: age at delivery, ethnicity, family history of DM, previous GDM, previous infant weighing ≥ 4500 g at birth, pre-gestational body mass index (BMI), delivery of a large for gestational age (LGA) infant, requiring insulin during GDM pregnancy, gestational age at delivery, and data about the six-weeks postpartum visit and laboratory evaluation.

Family history of DM was defined as having a first degree relative with type 2 DM. LGA was defined as a birth weight above the 90^th^ percentile, adjusted for gestational age, gender, parity, and ethnicity [8].

#### Questionnaire women

In August 2015, a letter was sent to all eligible women outlining the study goals and procedures, together with an informed consent form, a questionnaire, and a prepaid envelope. If needed seven weeks later a reminder was sent containing the same materials as the first invitation. The questionnaire comprised questions on educational level, breast feeding, and life style factors, including body weight, smoking, alcohol use, exercise, and diet.

Educational level was defined as low (primary education or intermediate vocational education), middle (higher secondary education), and high (higher vocational education and university). Duration of breastfeeding was labelled into four categories: 0 months, < 3 months, 3 to < 6 months, and ≥ 6 months.

Smoking status was defined as never smoker, ex-smoker or current smoker (1–6 or 6–20 cigarettes/day). Alcohol consumption was defined as ≤ 1 drinks/day (light drinker) and > 1–2 drinks/day (moderate drinker). Weight loss and weight gain were defined as a difference in weight of ≥ 5 kg compared to pre-pregnancy weight.

Exercise behaviour was assessed using the validated Short Questionnaire to Asses Health enhancing physical activity (SQUASH) questionnaire [9]. Women were asked to estimate commuting activities, leisure-time and sport-activities, household activities, and activities at work or school. According to the Dutch guidelines for healthy exercise, adults – 18 to 54 years – should have a moderate level of physical activity for at least half an hour, on at least five days a week [10].

According to the “Dutch guidelines for a healthy diet 2006” [11] we asked the women questions about their diet. These guidelines are translated by the Netherlands Nutrition Centre into the “Food Choice guidelines” and are formulated in terms of foods with two goals, to provide a nutritionally adequate diet containing all recommended macro- and micronutrients and to prevent chronic diseases [12]. The guideline contains five basic food groups which deliver the essential micro-and macro nutrients. These basic food groups are divided into three subgroups: foods with a positive, neutral, and negative effect on health. The subgroup criteria to classify foods are based on four nutrients that increase the risk of chronic diseases: saturated fatty acids, trans unsaturated fatty acids, added sugar, sodium, and one nutrient that decrease the risk: dietary fiber [12]. According to the basics foods groups and three subgroup criteria we asked women how much they eat of each basic food group and from which subgroup they mostly eat. For example: “How many parts of fruit do you eat per day?” “Which category is most consistent with your choice? A. Unprocessed fruit B. Pureed fruit or C. Fruit with added sugar/syrup”. The reported food choices were compared with the recommended amounts and the positive and neutral food groups were considered good [12]. The women were also asked about their knowledge of the “Food Choice guidelines” and their eating moments (“How many times per week do you eat breakfast, lunch, or dinner?” “How many times do you eat or drink per day?”). The Dutch Health Council advices seven eat/drink moments per day with a low intake of foods and drinks with easily fermentable sugars and drinks high in food acids (without the use of water, thee- and coffee (without sugar), or milk) [11].

#### Questionnaire general practitioners

Women were asked to give informed consent to send a questionnaire also to their GP. In November 2015, the eligible GP’s received a letter outlining the study, a questionnaire, and a copy of the informed consent form of their patient. If needed seven weeks later a reminder was sent containing the same materials as the first invitation. The questionnaire included questions about the annual follow-up screening. The GP was asked if he/she sent an annual reminder to their patient and if the patient has visited the annual postpartum controls (if the answer was no, “can you give a reason why your patient did not visit the annual postpartum controls”? If the answer was yes, “How many times did your patient visit the annual postpartum testing?”, “Did you repeat life style advices during the postpartum testing?”, and “Did your patient develop type 2 DM?”).

### Statistical analyses

All analyses were conducted using statistical package IBM SPSS Statistics (version 22.0. Armonk, NY: IBM Corp). Continuous data are given as mean ± standard deviation (SD) or as median and inter quartile range [IQR] in case of skewed distribution. Categorical data are given as number and percentage. Differences between the groups were tested using Student’s unpaired *t-*test for continuous data or Mann–Whitney *U* Test in case of skewed distribution. For categorical data Chi-square or Fisher’s exact test were used.

All *p*-values are two-tailed, and *p*-values below 0.05 were considered statistically significant.

## Results

**Fig. 1:**
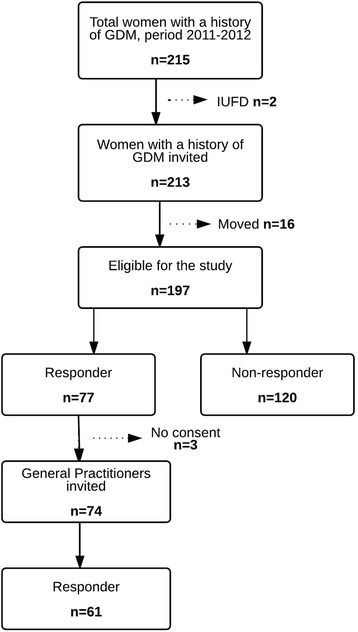
Flow-chart study population

In total 213 women were invited to participate in the study, 16 women had moved (Fig. 1). The most important clinical and demographic characteristic of the 197 eligible women (77 responders (39%), 120 non-responders (61%)) are summarized in Table 1. The women who responded were more often Caucasian (87%), and they more frequently had a previous infant weighing ≥ 4500 g at birth (14%). The women in the non-responder group had a slightly higher pre-gestational BMI and were more often obese (≥30 kg/m^2^) compared with the responder group. There were no differences in maternal age, family history of GDM, previous GDM, insulin requirements, LGA infant at delivery, and gestational age at delivery.

**Table 1:** Pre-gestational and gestational characteristics of the eligible study participants (period 2011–2012) according to survey responders and non-responders

Characteristics	Total	Responder		*P*-value*
		Yes	No	
N (%)	197	77 (39.1)	120 (60.9)	
Age at delivery (yrs)	32.2 ± 4.9	32.8 ± 5.0	31.9 ± 4.8	0.173
Family history of DM, n (%)	77 (41.8)	34 (47.2)	43 (38.4)	0.236
Previous GDM, n (%)	10 (5.1)	6 (7.8)	4 (3.3)	0.193
Previous infant weighing ≥ 4500 g at birth, n (%)	17 (8.6)	11 (14.3)	6 (5.0)	0.024
Pre-gestational BMI (kg/m^2^)	27.6 [24.1-31.2]	26.4 [22.9-30.6]	27.9 [24.3-32.0]	0.040
Pre-gestational BMI, n (%)				
< 25 kg/m^2^	61 (32.4)	27 (37.5)	34 (29.3)	0.236
25–29.9 kg/m^2^	61 (32.4)	25 (34.7)	36 (31.0)	
≥ 30 kg/m^2^	66 (35.1)	20 (27.8)	46 (39.7)	
Requiring insulin during pregnancy, n (%)	90 (45.7)	32 (41.6)	58 (48.3)	0.352
Gestational age at delivery (wks)	38.3 [38.0-39.6]	38.4 [38.0-39.7]	38.1 [37.7-39.5]	0.070
LGA infant, n (%)^‡^	41 (20.8)	16 (20.8)	25 (20.8)	0.993
Postpartum visit at the DM outpatient clinic, n (%)	156 (79.2)	71 (92.9)	85 (70.8)	0.001
Postpartum glucose testing, n (%)				
OGTT	62 (42.8)	31 (43.7)	31 (36.5)	0.558
HbA1c**	122 (61.9)	57 (74.0)	65 (53.3)	
SMBG	19 (13.1)	8 (11.3)	11 (12.9)	
Not test	11 (7.1)	3 (4.2)	8 (9.4)	
Discharge letter sent to the GP, n (%)	171 (86.8)	68 (88.3)	103 (85.8)	0.616
Copy of the discharge letter sent to women, n (%)	22 (11.2)	12 (15.6)	10 (8.3)	0.115

*Abbreviations*: *BMI* body mass index, *DM* diabetes mellitus, *GDM* gestational diabetes mellitus, *LGA* large for gestational age, *GP* general practitioner, *OGTT* oral glucose tolerance test, *SMBG* self-monitoring of the blood glucose

Data are expressed as mean ± SD, median [IQR], or proportion n (%)

Data with respect to family history of diabetes and pre-gestational body mass index are missing, in 13 (6.6%) and 9 (4.6%) of the women, respectively

**In 58 women who underwent a 75-g OGTT, Hb1Ac was also measured

^‡^Large for gestational age was defined as a birth weight above the 90^th^ percentile, adjusted for gestational age, gender, parity, and ethnicity

*P-values were based on Student’s unpaired *t*-test (non-skewed continuous variables), Mann–Whitney *U*-Test (skewed continuous variables) or chi-square test or fisher’s exact test (categorical variables)

Of the 197 eligible women, 156 (79%) attended the six-weeks postpartum office visit at the diabetes outpatient clinic in secondary care. More women in the responder group attended this postpartum office visit compared with the non-responders group. In 145 (93%) women who attended the postpartum visit glucose testing was performed. In total 62 (43%) women underwent a postpartum 75-g OGTT. Based on the postpartum 75-g OGTT, nineteen women had impaired glucose tolerance (2-h value after 75-g OGTT between ≥ 7.8 and 11.0 mmol/l) and one woman was diagnosed with type 2 DM. In 122 (84%) women HbA1c was measured. In 58 women who underwent a 75-g OGTT, HbA1c was also measured. In 19 (14%) women only SMBG was used to interpreted glucose status. HbA1c and SMBG were used for glucose status because of breastfeeding at time of the six-weeks postpartum visit (*n* = 58), patient declined the OGTT (*n* = 7), illness (*n* = 2), or the reason was unknown (*n* = 16).

Almost 87% of the GP’s received a discharge letter, 22 (11%) women received a copy of this discharge letter.

### Questionnaire women

Table 2 summarizes the follow-up lifestyle characteristics of the 77 women who responded to the questionnaire. The mean follow-up time was 3.5 ± 0.6 years. The median BMI was 26.5 [IQR 23.4-30.1] kg/m^2^ and is comparable with their pre-gestational BMI of 26.4 [IQR 22.9-30.6] kg/m^2^. More women lost weight (25%) (≥5 kg) than gained weight (20%) (≥5 kg) compared with their pre-gestational weight. Knowledge of the national recommendations for healthy exercise and the Dutch Food Choice guidelines was limited.

**Table 2:** Follow-up characteristics of the survey responders

Follow-up characteristics	*N* = 77
Postpartum (yrs)	3.5 ± 0.6
Age (yrs)	36.3 ± 5.0
Educational level, n (%)	
Low	7 (9.1)
Middle	33 (42.9)
High	37 (48.1)
Duration of breastfeeding, n (%)	
0 month.	24 (31.2)
< 3 months.	26 (33.8)
3 to 6 months.	11 (14.3)
> 6 months.	14 (18.2)
Current smoker, n (%)	17 (22.1)
Alcohol consumption, n (%)	
Non-drinker	29 (37.8)
≤ 1 drink/d	46 (59.7)
> 1–2 drinks/d	2 (2.6)
Weight	
BMI (kg/m^2^)	26.5 [23.4-30.1]
BMI, n (%)	
< 25 kg/m^2^	24 (31.2)
25–29.9 kg/m^2^	29 (37.7)
≥ 30 kg/m^2^	21 (27.3)
Weight gain, n (%)^†^	15 (19.5)
Weight loss, n (%)^†^	19 (24.7)
Physical Activity	
Knowledge of Dutch recommendations on physical activity, n (%)	12 (15.6)
Active 30 min 5 days/week, n (%)	27 (35.1)
Diet	
Knowledge of Dutch Food choice guidelines, n (%)	
Yes	40 (51.9)
No, not exactly	31 (40.3)
No, never heard of it	6 (7.8)
Eating three meals p/d, n (%)	55 (71.4)
Max. two snacks p/d, n (%)	67 (87.0)
Fish two times p/wk, n (%)	15 (19.5)
Meeting the recommendations for basic food groups	
Fruit, n (%)	27 (35.1)
Vegetables, n (%)	6 (7.8)
Bread and grain products, n (%)	1 (1.3)
Potatoes, rice, pasta, legumes, n (%)	5 (6.5)
Milk (products), n (%)	28 (36.4)
Cheese, n (%)	43 (55.8)
Meat, fish, poultry, eggs, meat substitutes, n (%)	10 (13.0)
Oils and soft margarines, n (%)	25 (32.5)
Drinks, n (%)	27 (35.1)

*Abbreviations*: *BMI* body mass index. Data are expressed as mean ± SD, median [IQR], or proportion n (%)

Data with respect to body mass index and breastfeeding are missing, in 3 (3.9%) and 2 (2.6%) of the women, respectively. ^†^Weight loss since pregnancy was defined as ≥ 5 kg and weight gain as ≥ 5 kg

### Questionnaire physician’s

Outcomes of the annual follow-up glucose testing at the GP are summarized in Table 3. In total 74 GP’s were invited to participate in the study, and 61 GP’s (82%) responded. Of the 61 GP’s, 12 GP’s reported that they performed follow-up glucose testing within >12–14 months after delivery. Only two women were tested for three consecutive years. Reasons GP’s provided for not screening are also summarized in Table 3.

**Table 3:** Annual glucose screening at the general practitioner

GP responders	*N* = 61*
Annual postpartum control, n (%)	*N* = 12 (20.0)**
Received a discharge letter from secondary care, n	11
Patient in a re-call system, n	4
Total number of annual glucose screenings, n	
1 year follow-up	5
2 years follow-up	5
3 years follow-up	2
Provided lifestyle advices, n	4
Total women diagnosed with type 2 diabetes, n	1
No annual postpartum control, n (%)	*N* = 49 (80.0)***
Received a discharge letter from secondary care, n	43
Patient in a re-call system, n	15
Reasons for not screening, n	
Patient declined testing	1
GP did not know testing is needed	4
Patient not in a call system	1
GP did not see the patient, despite a reminder	4
Patient had a second pregnancy within 1 year	2
Patient had other GP at time of GDM pregnancy	4
Controls at endocrinologist	5
Reason unknown	28

*Abbreviations*: *GP* general practitioner, *GDM* gestational diabetes mellitus

Data are expressed as proportion, n (%)

*Total general practitioners responded

**Total general practitioners responded and who screened their patient within 12–14 months after delivery

***Total general practitioners responded and who did not screened their patient within 12–14 months after delivery

## Discussion

In this follow-up survey of women with a history of GDM and their GP, we found high rates of the six-weeks postpartum visit and glucose testing at the diabetes outpatient clinic. However, we found low rates of longer-term follow-up postpartum glucose testing in primary care.

Moreover, we found suboptimal performance of adherence to a healthy lifestyle for women with a history of GDM, particularly with respect to exercise and to a lesser extent regarding diet. Most of the GP’s also did not provide lifestyle advices at the annual follow-up glucose testing.

During the six-weeks postpartum office visit a noteworthy number of women tested with a 75-g OGTT were found to have impaired glucose tolerance and one woman was diagnosed to have type 2 DM. This finding indicates that a number of women are in an advanced stage of developing type 2 DM. Studies suggest that the association between GDM and type 2 DM can be explained by the fact that many of the risk factors for both disorders are the same, including high BMI, family history of diabetes, and ethnic origin [2, 3]. The prevalence of GDM is increasing worldwide and former GDM women are for several years at risk to develop type 2 DM. Therefore, the low long-term rates are a missed opportunity to postpone obesity and type 2 DM, disease which carry a high burden for the individual patient and for society.

There is limited published data on the long-term follow-up testing in primary care of women who have had GDM. Two recent studies [13, 14], conducted in the United Kingdom, investigated the long-term follow-up testing of women with a history of GDM. The first study demonstrated that during a 5-year period around 20% of the 718 women with a history of GDM had long-term follow-up glucose testing and only three (0.4%) women were followed-up every year [14]. Another study showed that of the 233 included women, 34% had glucose testing in the first year postpartum, 12% (16 of the 131) in the second year and 18% (8 of the 45) 3 years after delivery [13]. These findings are in line with the rates of long-term follow-up glucose testing found in our study.

There are several potential explanations for the low long-term follow-up postpartum testing rates. This study indicates that follow-up of women with GDM is insufficiently incorporated in the primary care system. Possible reasons for this hampered follow-up system may include a lack of agreed protocols, insufficient or unclear communication by the treating physician in secondary care, and a lack of sufficient call- and tracking systems in Dutch family practices. Although the Dutch Society of Obstetrics and Gynaecology guideline 2010 “Diabetes and Pregnancy” recommends glucose testing six-weeks after delivery and subsequently once a year for the next 5 years, such follow-up glucose testing was added to GP guidelines only in 2013 [15].

There may be limited knowledge and reduced awareness of the importance of postpartum screening among GDM women. A number of GP’s reported that also the women declined testing and did not respond to a follow-up invitation despite sending a reminder. This is remarkable as during the six-week postpartum visit, all women have verbally received information about their future risk of type 2 DM and they were instructed to visit their GP at least annually for follow-up glucose testing. However, only a small number received a copy of the discharge letter sent to their GP, which summarized these verbal communications, and none have received additional written information on the risk of development of type 2 DM and adoption of a healthy lifestyle. Studies have demonstrated that lifestyle modifications including weight loss, healthy diet, and moderate exercise can reduce the risk of developing type 2 DM in high risk subjects [16, 17] and also in women with a history of GDM [18–20]. For this reason, during the six-weeks postpartum visit all women with a history of GDM received information about the benefits of weight management and moderate physical activity. In this study all women received a life-style questionnaire to examine the life-style of women with a history of GDM. The women who responded at the questionnaire were less often obese, had higher rates of a previous infant weighing ≥4500 g at birth, and more women attended the six-weeks postpartum visit and glucose testing compared with the non-responder group. These findings, may suggest that the responders group is more interested and aware about the importance of lifestyle changes compared with the non-responder group. However, also in the responder group only one third of the women met the recommendations of physical activity and there were suboptimal levels of dietary intake, for instance a low intake of fruit and vegetables. Moreover, most of the women were overweight (BMI ≥25-29.9 kg/m^2^) or obese (BMI ≥30 kg/m^2^). A positive finding was the fact that more women lost weight than gained weight compared with their pre-pregnancy weight. A few studies have investigated the health status and lifestyle modifications in women with a history of GDM [21–27]. In analogy to our study, these studies showed that diet and physical activity levels rarely met the recommendations [22, 23, 26, 27].

Even though most of the women were highly educated, we found a suboptimal performance of adherence to a healthy lifestyle. There are several barriers for women with a history of GDM to adopt a healthy lifestyle including time, financial constraints, child care, lack of motivation, and lack of social support [28]. These barriers are also found in women with the same age without a history of GDM [22].

In the Dutch health-care system, the GP could be a good motivator for women to adopt a healthier lifestyle [29]. However, the GP’s did not reinforce a healthier lifestyle as most of them did not provide such advices at the annual follow-up. This study and previous studies indicated that there is need for better lifestyle awareness and coaching in primary care, including advice about diet and physical activity. With our current health-care system we are missing opportunities to interfere in obesity and type 2 DM development. Successful monitoring depends on a good implementation in secondary and primary care and education of self-management and adherence of the patient. There are several recommendations for the organization of care to expand and improve the long-term follow-up in former GDM women.

First, the internist can improve care by sending a discharge letter to the GP and a copy of the letter to the patient, including written information (a brochure) about the future risk of type 2 DM. Secondly, the GP can denote a previous GDM patient carrying an increased cardiovascular risk and connect them to a tracking system in the GP practices and send postnatal reminders to women. A study in South Australia has shown that a GDM Recall Register for former GDM women – sending a reminder 15 months after the expected delivery date - is successful in recruiting women to remind them that they should continue to have their blood glucose checked over the long term [30]. At last, another place for health promotion and raise further awareness could be the Early Childhood Centers. Women visit the Early Childhood Center regularly with their baby’s (0–4 years), and their nurses can help to recommend the mother to pursue follow-up care by their GP. Regional lifestyle programs/self-management programs can be offered by the Early Childhood Centers, for example in group classes.

### Strengths and limitations

Strengths of the study are the evaluation of postpartum glucose visits in secondary care and additional the annual long-term postpartum glucose testing in primary care. To our knowledge this is the first study in the Netherlands which investigated the glucose screening of women with a history of GDM.

This study has several potential limitations. This study was conducted at only one institution with a relative small sample size. Therefore, the total number of women with a history of GDM who responded was too low to evaluate the type 2 DM incidence. Furthermore, we have no information about lifestyle factors of the women before their pregnancy. For this reason, we cannot determine to what extent the educational issues connected to GDM motivated patients to change lifestyle for example lose weight. Finally, in most of the guidelines an OGTT is recommended for blood glucose testing, because the OGTT has a high sensitivity compared with other screening methods. In our national guideline the OGTT is not the standard for blood glucose screening postpartum, because the OGTT screening method is time consuming and not patient-friendly.

## Conclusions

In conclusion, this study demonstrated that in women with a history of GDM postpartum follow-up care was far from optimal and showed a striking discrepancy with current guidelines. Long-term postpartum follow-up clearly requires improvements in the Netherlands, to early diagnose pre-diabetes or type 2 DM and more importantly to pay attention to preventive strategies. The improvement of long-term follow-up testing could be realized by marking GDM patients and connect them to a yearly recall-system. Also a better communication between primary and secondary care is needed. Finally, awareness among women with a history of GDM will probably surface a healthier lifestyle and at least decrease the low rates at the postpartum visit in primary care. There is clearly more need for lifestyle coaching programs/self-management for women with a history of GDM to adopt a healthy lifestyle and make them aware about the risk of type 2 DM.

## Abbreviations

BMI: Body mass index
DM: Diabetes mellitus
GDM: Gestational diabetes mellitus
GP: General practitioner
IQR: Inter quartile range
LGA: Large for gestational age
OGTT: Oral glucose tolerance test
SD: Standard deviation
SQUASH: Short questionnaire to asses health
